# Suspended hydrogel culture as a method to scale up intestinal organoids

**DOI:** 10.1038/s41598-023-35657-9

**Published:** 2023-06-27

**Authors:** Julia Y. Co, Jessica A. Klein, Serah Kang, Kimberly A. Homan

**Affiliations:** Complex in vitro Systems, Safety Assessment, Genentech, 1 DNA Way, South San Francisco, CA 94080 USA

**Keywords:** High-throughput screening, Gastrointestinal models, Stem-cell biotechnology

## Abstract

Primary tissue-derived epithelial organoids are a physiologically relevant in vitro intestinal model that have been implemented for both basic research and drug development applications. The existing method of culturing intestinal organoids in surface-attached native extracellular matrix (ECM) hydrogel domes is not readily amenable to large-scale culture and contributes to culture heterogeneity. We have developed a method of culturing intestinal organoids within suspended basement membrane extract (BME) hydrogels of various geometries, which streamlines the protocol, increases the scalability, enables kinetic sampling, and improves culture uniformity without specialized equipment or additional expertise. We demonstrate the compatibility of this method with multiple culture formats, and provide examples of suspended BME hydrogel organoids in downstream applications: implementation in a medium-throughput drug screen and generation of Transwell monolayers for barrier evaluation. The suspended BME hydrogel culture method will allow intestinal organoids, and potentially other organoid types, to be used more widely and at higher throughputs than previously possible.

## Introduction

Intestinal epithelial organoids are 3D multicellular spheroids that recapitulate the complexity and functions of the intestinal tissue in vivo, and have emerged as a physiologically relevant in vitro model of the intestine. These intestinal organoids, also termed “enteroids” or “colonoids”, are derived from adult stem cells isolated from primary intestinal tissue, and can be easily propagated and cryopreserved for long term storage. Intestinal organoid cells can differentiate into various intestinal cell types, perform epithelial functions such as barrier maintenance, absorption, secretion, and digestion, and recapitulate biological characteristics and clinical responses of the patients from which they are derived^1,2^. Thus, intestinal organoids have been widely adopted in place of traditional transformed and immortalized intestinal cell lines, leading to basic science discoveries and facilitating translational applications in numerous fields including cancer biology, infectious diseases, and cystic fibrosis^2,3^.

A key challenge that has limited implementation of intestinal organoids in areas such as drug development is that the current organoid culture technique is difficult to scale up, requiring tedious manual approaches or development of advanced automation infrastructure^4^. In the existing method, intestinal epithelial stem cells (either isolated from primary tissue or passaged from established organoid cultures) are resuspended in a cold extracellular matrix (ECM), most often Cultrex Basement Membrane Extract (BME) or Matrigel hydrogels. The ECM-cell solution is deposited onto the surface of a plate (typically a 24-well or 6-well plate), then warmed to cure into a surface-attached hydrogel dome, and overlaid with media. Media in each well is changed every few days and intestinal organoids form over the course of 1–2 weeks^5–8^. This technique is difficult to scale up because it is limited by available surface area for hydrogel dome formation, it is time-consuming, labor-intensive and can be prone to user error, and diffusion limitations of the hydrogel cause heterogeneity in organoid growth and morphology^9–11^.

We have developed a simplified intestinal organoid culture method that enables large-scale organoid culture without tedious manual handling, specialized equipment, or automation. By growing organoid cells in suspended ECM hydrogels instead of the conventional surface-attached hydrogel domes, the hydrogel volume and thus the organoid cells that can grow in a culture vessel can be drastically increased. Because it does not require hydrogels to be deposited on a 2D surface, this method is compatible with culture flasks, which allows further culture scale-up. The method encompasses varying geometries of suspended ECM hydrogels, which can expedite the hands-on culture preparation time. Moreover, because the organoids are in suspension, they can be sampled, divided, or collected at various times during culture, which is difficult for conventional cultures immobilized in a plate. Organoids in suspended BME hydrogels grow more uniformly than in conventional surface-attached hydrogel domes, where limited molecular diffusion results in nutrient gradients^9,10^. We demonstrate that our scalable culture method yields organoids that are comparable to those cultured in the conventional method and provide examples of downstream applications including a drug toxicity screen and generation of a Transwell monolayer model for barrier evaluation. Overall, the suspended BME hydrogel culture method will make intestinal organoid models more amenable to high-throughput studies that will benefit both basic science and translational fields.

## Results

### A suspended BME hydrogel method for human intestinal organoid culture

**Figure 1:**
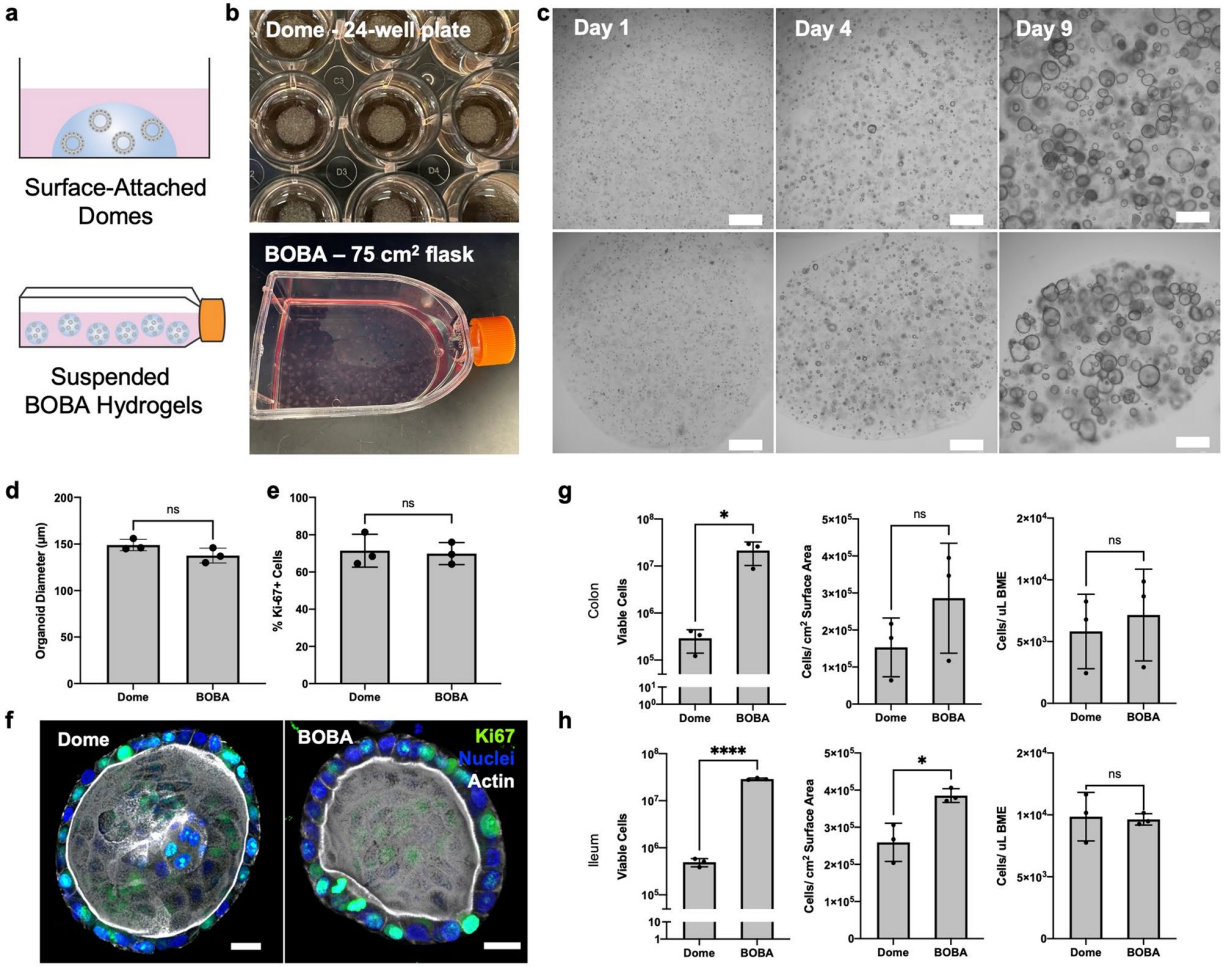
Scale-up of intestinal organoids in suspended BME hydrogels. (**a**) Schematic, (**b**) photographs, and (**c**) brightfield microscopy of human colon organoids in conventional Dome culture (top) and suspended BOBA (BME-embedded Organoid Bead Assembly) culture (bottom). Scale bars for micrographs are 500 µm. (**d**) Organoid diameters for dome and BOBA cultures in brightfield images. (**e**) Percent Ki67-positive cells in confocal images. Data represented are mean ± SD, Student’s t-test, n = 3 with 10 fields of view each. (**f**) 3D-reconstructed confocal images of colon organoids in Dome or BOBA cultures. Ki67 in green, nuclei in blue, actin in white, scale bars are 20 µm. (**g**, **h**) Total viable cells, cells per cm^2^ surface area, or cells per µL BME for (**g**) colon and (**h**) ileum organoids. All data represented are mean ± SD, Student’s t-test *p ≤ 0.05,****p ≤ 0.0001, n = 3 experiments.

The conventional intestinal organoid culture method requires that a solution of organoid cells in cold ECM is deposited onto a plastic surface, cured in an incubator to form a hydrogel dome, then overlaid with growth media (Fig. 1a, b)^5–8^. To address the limitations of scaling this technique up, we developed a method in which cold cell-ECM solutions are cured instantly as floating hydrogels suspended in warm media. We demonstrate the method using suspended droplets of BME, an Engelbreth-Holm-Swarm (EHS) cell-derived ECM equivalent to Matrigel, and have termed the method BOBA (BME-embedded Organoid Bead Assembly) culture.

We first evaluated intestinal organoid growth in the suspended BOBA culture method compared to the conventional surface-attached BME dome method, referred to here as Dome culture. For the Dome culture, single cells from digested organoids were suspended in cold BME and plated as 50 µL domes in a 24-well plate, cured for 15–30 min in a 37 °C incubator, then overlaid with growth media (Intesticult Organoid Growth Medium). Media changes were performed individually for each well every 2–3 days. For the BOBA culture, the single cell-BME solution was deposited using an electronic repeater pipette with wide-bore tips as 10 µL droplets directly into pre-warmed media, where the hydrogel cured immediately into suspended BOBAs. The BOBAs could be generated in petri dishes, plates, or conical tubes, and were easily transferred to larger vessels like cell culture flasks via serological pipette or decanting (Fig. 1a, b). Media changes were performed for an entire flask by allowing BOBAs to settle, then replacing the top 75% volume of spent media with fresh media.

Human intestinal organoid cell-BME solutions were plated in parallel Dome or BOBA cultures and grown for 9 days in growth media. Organoid growth and size were similar in both methods as observed by brightfield microscopy (Fig. 1c) and quantified with organoid diameter measurements (Fig. 1d). Organoid cell proliferation was also comparable, as determined by quantifying the abundance of cells expressing proliferation marker Ki-67 (Fig. 1e, f). The BOBA method appeared to enable more organoid cell growth per cm^2^ surface area, although we observed statistical significance only for small intestine (ileum) organoids (Fig. 1g, h). For colon organoids, Dome culture yielded a mean of 2.9 × 10^5^ viable cells per well or 1.5 × 10^5^ cells/cm^2^ in a 24-well plate, while BOBA culture yielded a mean of 2.2 × 10^7^ viable cells or 2.9 × 10^5^ cells/per cm^2^ in a 75 cm^2^ flask (Fig. 1g). For ileum organoids, Dome culture yielded a mean of 4.8 × 10^5^ viable cells per well or 2.6 × 10^5^ cells/cm^2^ in a 24-well plate, while BOBA culture yielded a mean of 2.9 × 10^7^ viable cells or 3.9 × 10^5^ cells/per cm^2^ in a 75 cm^2^ flask (Fig. 1h). The number of viable cells per µL BME hydrogel was similar, indicating that the amount of growth given a fixed seeding density is comparable for both methods (Fig. 1g,h).

### Organoid differentiation in BOBA culture

**Figure 2:**
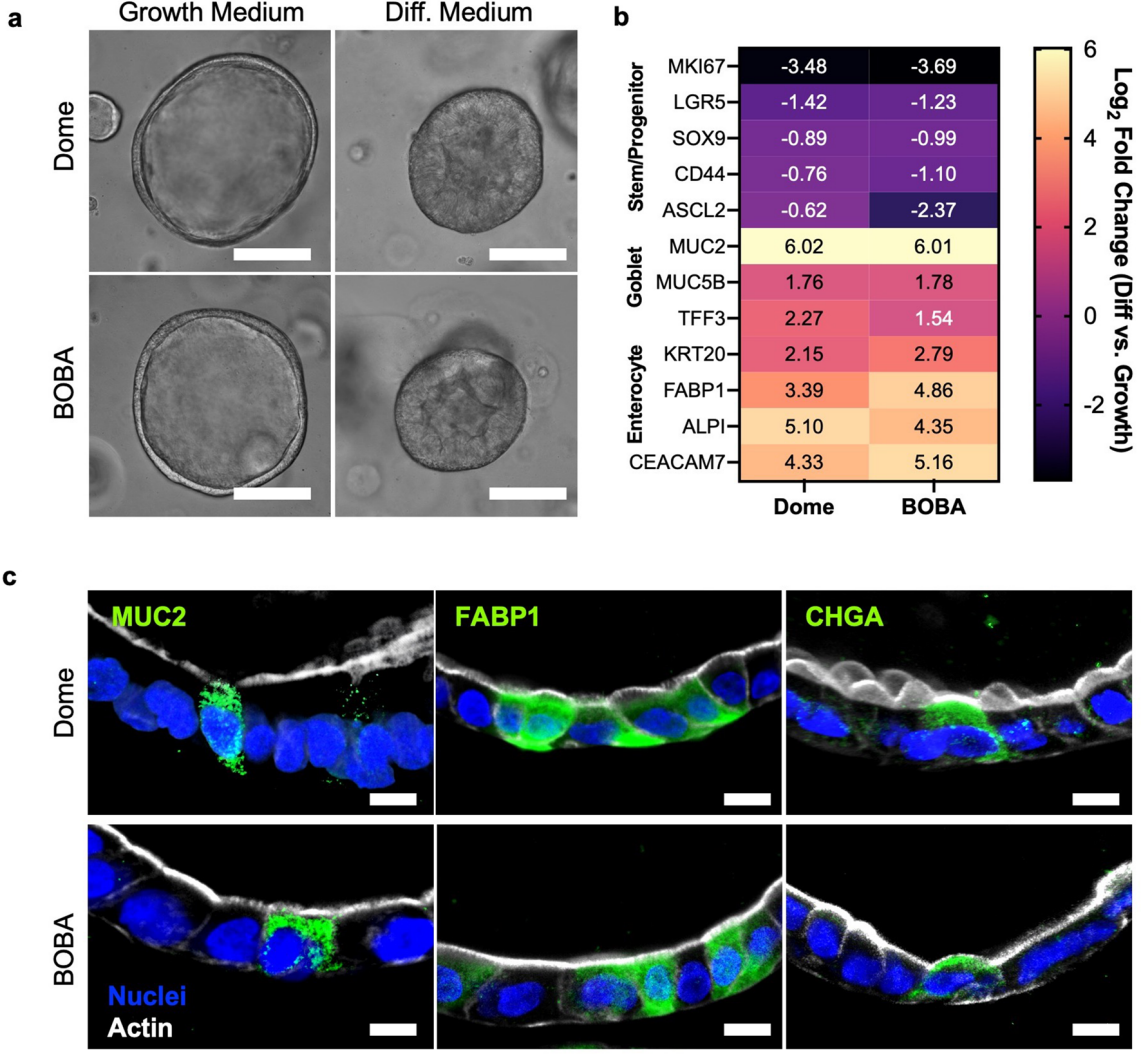
Organoid differentiation in BOBA culture. (**a**) Brightfield images of colon organoids in Growth Media or Differentiation Media show that organoids cultured using the BOBA method have comparable morphologies to those cultured using the Dome method. Scale bars are 100 µm. (**b**) Bulk RNA-seq shows that Dome- and BOBA-cultured colon organoids similarly downregulate stem and progenitor markers and upregulate differentiation markers after transition from Growth Media to Differentiation Media. (**c**) Confocal microscopy shows that markers of differentiated epithelial cell types (MUC2 for goblet cells, FABP1 for enterocytes, and CHGA for entero-endocrine cells) are expressed in organoids cultured in both Dome (top) and BOBA (bottom) formats. Nuclei in blue, Actin in white, scale bars are 10 µm.

A key benefit of the intestinal organoid model is the ability to differentiate into the various intestinal epithelial cell types by altering the media composition, for example by withdrawing stem cell-promoting factors^1–3^. We compared differentiation of organoids in Dome and BOBA cultures. Organoids were grown in growth media for 7 days, then washed and transitioned to differentiation media (Intesticut Organoid Differentiation Medium with 5 µM DAPT) for 5 days. Brightfield microscopy showed that in both culture formats, organoids in growth media exhibited a cyst morphology with a large lumen (Fig. 2a) and organoids in differentiation media exhibited a dense spheroid morphology with elongated columnar cells and a small lumen (Fig. 2a).

Bulk RNA-seq analysis was performed for organoids in growth media or differentiation media, in Dome and BOBA culture. In both culture methods, organoids in differentiation media exhibited downregulated expression of stem cell and proliferation markers (*MKI67*, *LGR5*, *SOX9*, and *CD44*) and upregulated expression of differentiation markers for goblet cells (*MUC2*, *MUC5B*, *TFF3*) and enterocytes (*KRT20*, *FABP1*, *ALPI*, and *CEACAM7*) relative to organoids in growth media (Fig. 2b, S2a), and organoids grown in growth media and differentiation media clustered separately in the principal component analysis (PCA) plots (Fig. S2b). Differentiated cell types were also observed by immunofluorescence (IF) confocal microscopy in both culture formats (Fig. 2c).

### Characterization and optimization of BOBA culture conditions

We next evaluated how varying culture parameters would impact organoid growth in the BOBA method. Cultures were seeded in either 6-well plates (Fig. S1a, b) or 25 cm^2^ flasks (Fig. S1c), with various volumes of BME and a fixed cell seeding density of 6 × 10^5^ cells/mL of BME. All BOBAs were generated in 10 µL droplets with 5 mL of growth media, and organoid growth was evaluated by quantifying organoid diameters and viable cell numbers after 9 days of culture (Fig. S1b, c). In 6-well plates, as the total BME volume per well increased from 0.5 to 2 mL, the organoid diameters decreased and the ratio of viable cells to volume BME decreased (although not statistically significant). The total number of viable cells and ratio of viable cells per cm^2^ surface area was comparable for all conditions, despite a higher number of seeded cells in the higher BME volume conditions, indicating that there was less proliferation per cell seeded. Together, these data suggest that organoid growth is impaired when the BME volume exceeds a threshold for a fixed amount of media in a 6-well plate (Fig. S1B).

Interestingly, for BOBA cultures in 25 cm^2^ flasks, organoids grew equally well in all tested conditions (Fig. S1c). As the total BME volume per flask increased from 0.5 to 2 mL, the organoid diameters and the ratio of viable cells to volume BME were similar. The total number of viable cells and ratio of viable cells per cm^2^ surface area increased as the volume of BME in the culture appeared to increase (although not statistically significant due to experiment-to-experiment variability), suggesting that the threshold for ratio of BME to media is different for the 6-well plate and 25 cm^2^ flasks. Both BME-to-media ratio and vessel type should be optimized for specific applications.

### Organoid uniformity in BOBA culture

**Figure 3:**
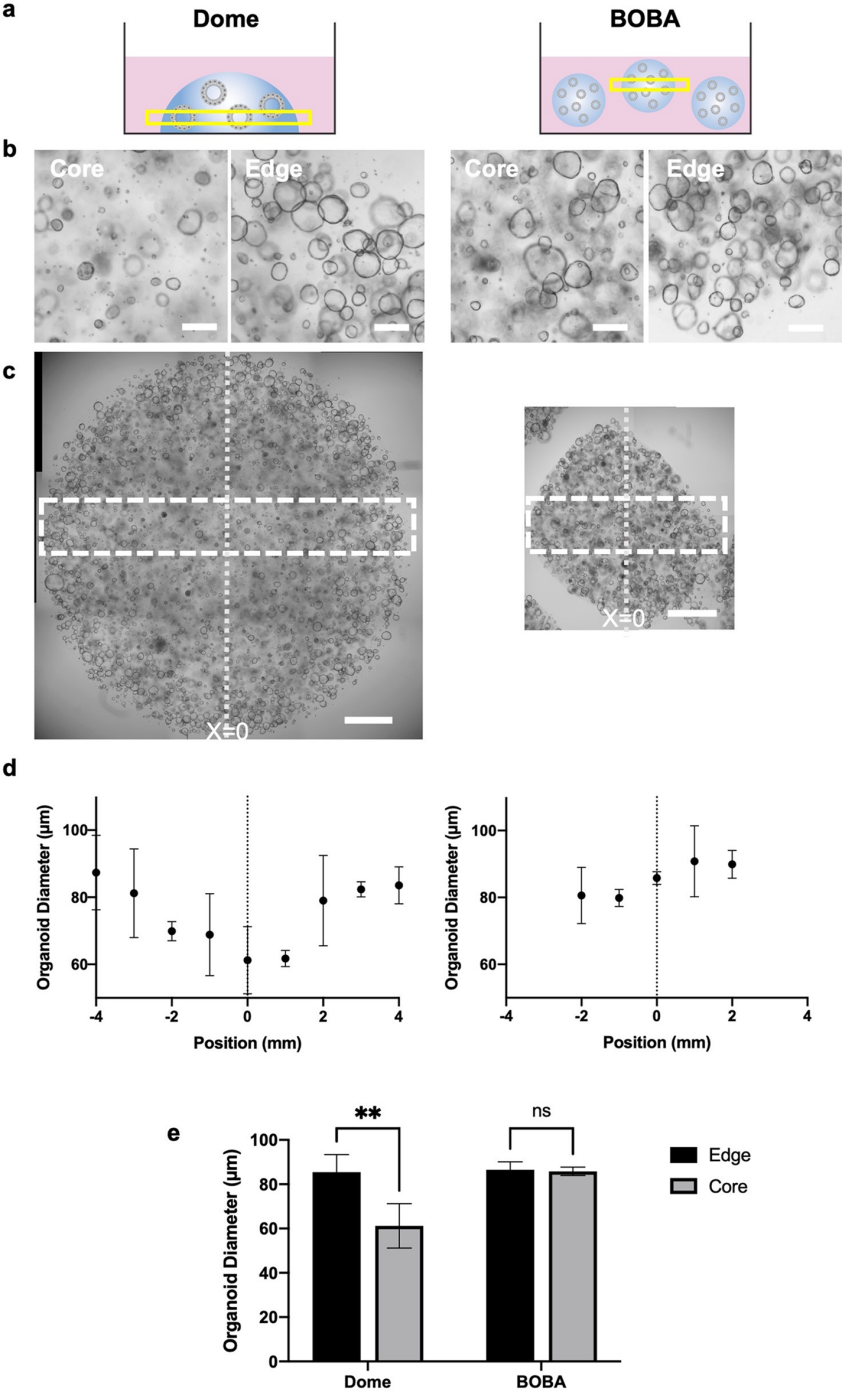
Organoid size uniformity in BOBA cultures. (**a**) Schematic describing imaging strategy for uniformity analysis. (**b**, **c**) Brightfield images of colon organoids at the deepest plane in a 50 µl BME dome or a 10 µl suspended BOBA hydrogel. Scale bars are (**b**) 200 µm and (**c**) 1 mm. (**d**) Quantification of organoid diameter in a horizontal ROI of a Dome or BOBA hydrogel by position across the X-axis. Data represented are mean ± SD, n = 3 replicates in a representative experiment of 3 experiments. (**e**) Quantification of mean organoid diameter at the edge or core of Dome and BOBA cultures. All data represented are mean ± SD, Two-way ANOVA, n = 3 replicates in a representative experiment of 3 experiments.

The conventional Dome method is known to cause organoid heterogeneity^5,11^. Native ECM hydrogels limit gas and molecular diffusion, resulting in nutrient gradients and heterogeneous organoid growth^9,10,12^. We observed that organoids grow more uniformly in BOBA culture than in Dome culture (Fig. 3). Colon organoid cultures were imaged by brightfield microscopy at the deepest point in each format—the bottom z-plane of Domes or the center z-plane of BOBAs—and the mean organoid diameter across a rectangular ROI in the center of each hydrogel was quantified (Fig. 3c). In agreement with previous reports^9,10^, Dome culture organoids grew larger at the hydrogel edges and smaller at the core (Fig. 3b–e). However, organoids in BOBAs grew to comparable sizes across the hydrogel (Fig. 3b–e).

We also observed that the organoids in the core of the dome culture often had the dense spheroid small-lumen or lumen-less morphology that often indicates differentiation (Fig. 3b). Supporting the hypothesis that there may be a subpopulation of differentiated organoids in Dome cultures, bulk RNA-seq analysis indicated that Dome culture organoids had lower expression of stem cell and proliferation markers and higher expression of enterocyte markers relative to their BOBA culture counterparts (Fig. S3). These differences may explain the separate clustering of the Dome and BOBA samples grown in growth media in the PCA plot (Fig. S2B).

### Alternative geometries for suspended BME hydrogels

While the BOBA method provides significant scale-up advantages over the conventional Dome method, massive culture expansion of suspended BOBA hydrogel droplets without an automated liquid handler can still be a labor-intensive endeavor. To reduce time and labor required for suspended BME hydrogel culture, we devised an alternate hydrogel geometry, specifically hydrogel filaments. Extruded filaments have been employed in the bioprinting field to spatially control cell growth or to build layer-by-layer assemblies of 3D hydrogel structures, but typically rely on attachment to a surface^13,14^. To generate an organoid cell-containing hydrogel filament, termed SOBA (Syringe-extruded Organoid BME Assembly), a cold cell-BME solution was loaded into a syringe with a 15-gauge (1.37 mm inner diameter) blunt-tip needle, then injected directly into warm media while moving the tip across the X–Y plane (e.g. in a linear, snake, or spiral pattern). We also generated filament fragments which more closely resemble the BOBA droplet geometry, termed SOBA fragments, by gentle trituration of SOBA cultures with a wide bore P1000 tip or a 10 mL serological pipette.

**Figure 4:**
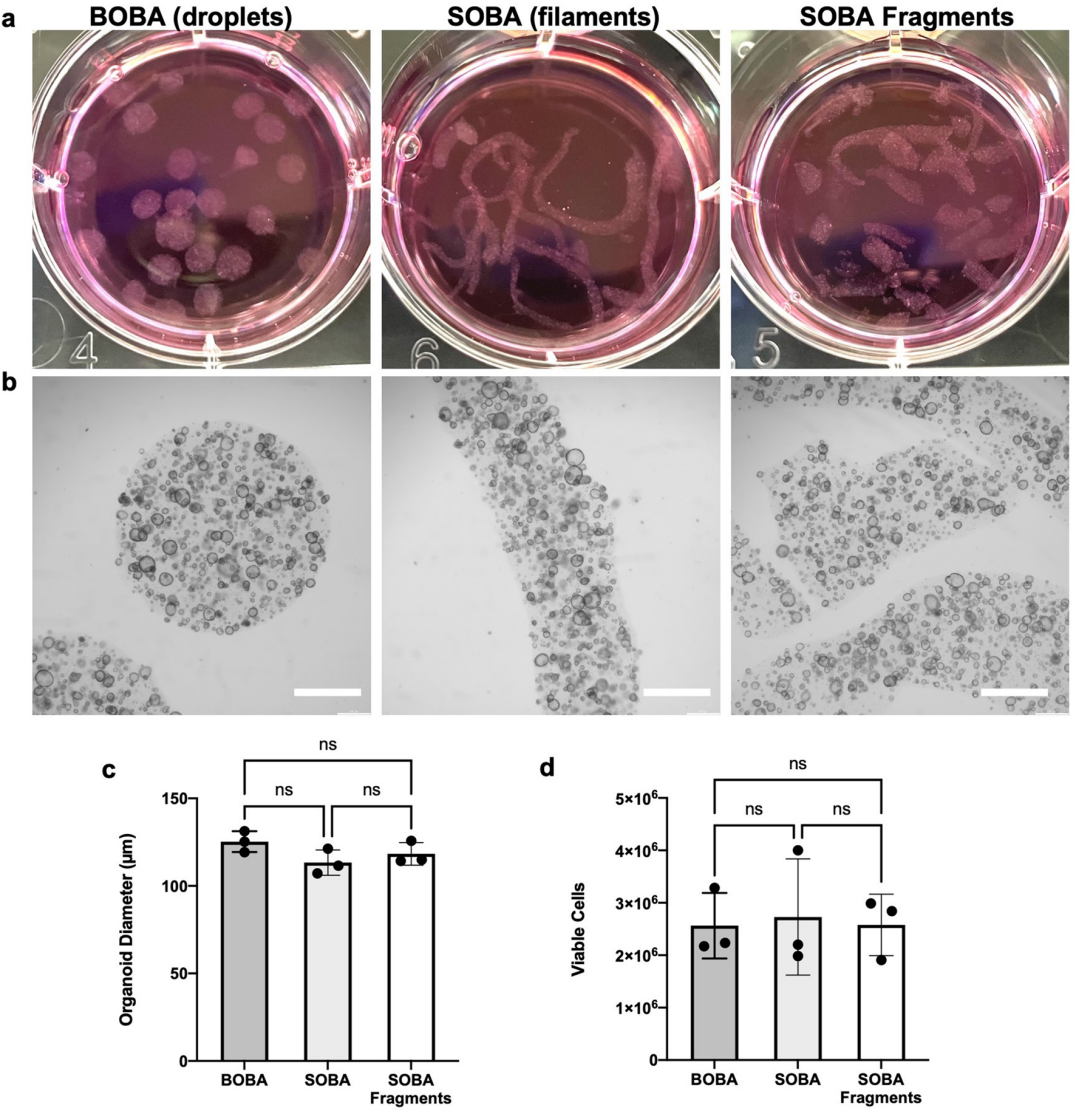
Alternate suspended BME hydrogel culture formats show comparable organoid growth. (**a**) Photographs and (**b**) brightfield images of colon organoids in 6-well plate cultures in BOBA, SOBA, or SOBA fragment formats. Alternate formats enable expedited culture preparation for scale-up. Scale bars are 1 mm. (**c**) Quantification of organoid diameter and (**d**) viable cells per well. Data represented are mean ± SD, One-way ANOVA multiple comparison test, n = 3 experiments.

Organoids grown in BOBA, SOBA, or SOBA fragment cultures for 9 days showed similar growth determined by brightfield microscopy (Fig. 4a), organoid diameter measurements (Fig. 4b, c) and viable cell counts (Fig. 4d). Relative to BOBA droplets, SOBA, and SOBA fragment formats yield comparable organoid growth while enabling faster and less labor-intensive culture preparation. Compared to SOBA cultures, SOBA fragments are more evenly dispersed in the media, thus allowing a single culture to be more easily divided, sampled, or aliquoted.

### Application of suspended BME hydrogel organoids in a drug toxicity screening assay

**Figure 5:**
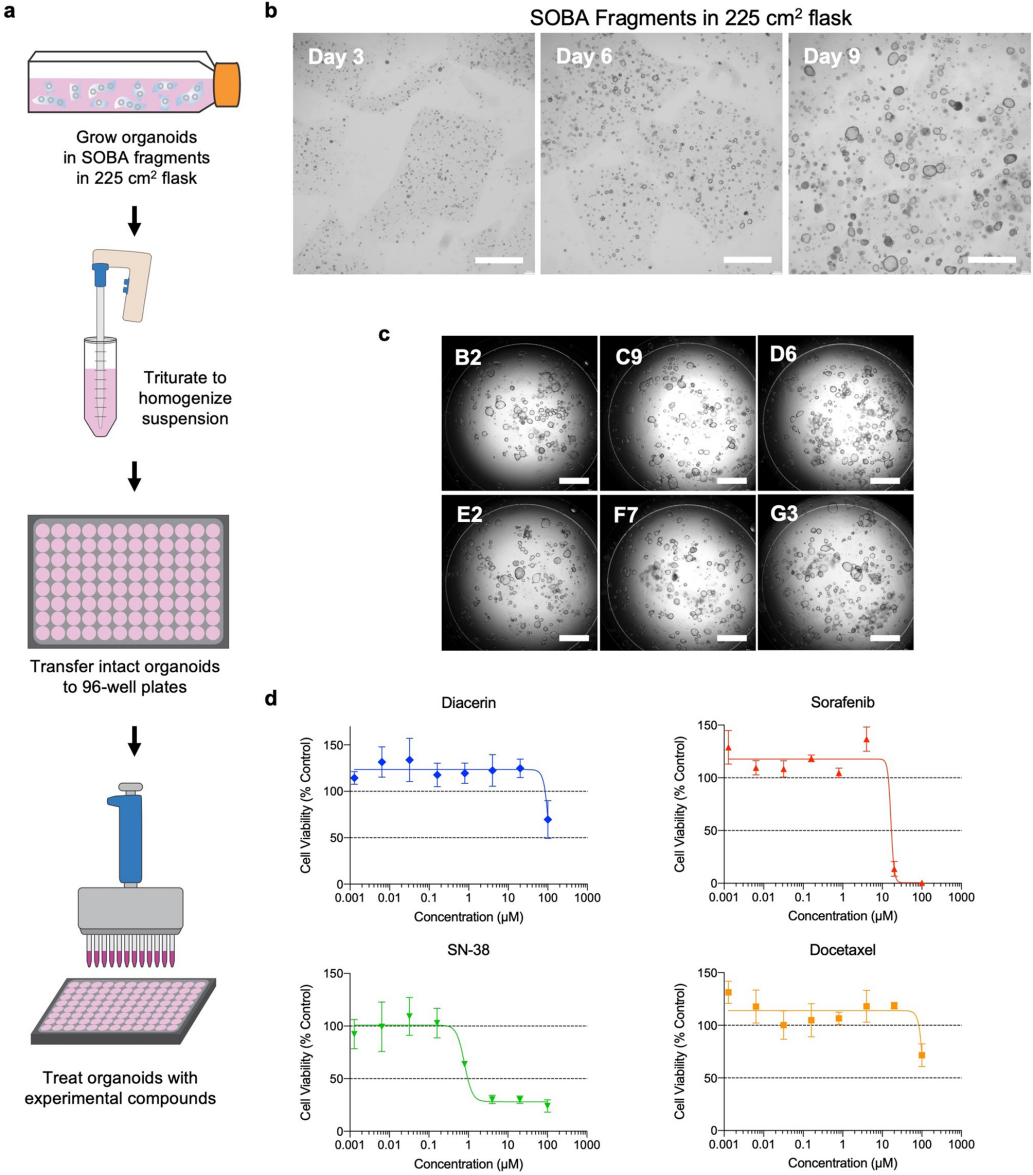
Application of suspended BME hydrogel organoid cultures in a medium-throughput screen. (**a**) Schematic of experiment. SOBA fragment colon organoid culture in a 225 cm^2^ flask was triturated to achieve a uniform organoid suspension. The organoid suspension was seeded in 96-well plates, then treated with experimental compounds. (**b**) Brightfield images of a SOBA fragment culture cultured in a 225 cm^2^ flask. Scale bars are 1 mm. (**c**) Brightfield images of wells chosen at random across a 96-well plate show comparable well-to-well organoid densities. Scale bars are 1 mm. (**d**) Representative dose response viability curves (Cell Titer Glo 3D assay) for suspended BME organoids treated with diacerin, sorafenib, SN-38, or docetaxel for 3 days. Data represented are mean ± SD, n = 4 wells per condition.

Intestinal organoids generated in BOBA, SOBA or SOBA fragment formats can be used directly in downstream assays without additional organoid digestion or passaging steps. As a proof of concept, we demonstrate the implementation of these organoids in a drug toxicity screen. SOBA fragment-cultured colon organoids were grown in a 225 cm^2^ flask (Fig. 5a, b), homogenized by trituration with a serological pipette, then transferred to 96-well plates (Fig. 5a, c). Well-to-well variability of 96 wells in a 96-well plate, measured by the Cell Titer Glo 3D (CTG) ATP-based viability assay, was comparable for SOBA fragment-grown organoids (SD = 0.168) and Dome cultures plated in a 96-well plate (SD = 0.205) Fig. S4.

For the drug toxicity screen, SOBA fragment-grown organoids in the 96-well plates were treated with compounds known to cause intestinal toxicity (diacerin, sorafenib, SN-38, or docetaxel) or a DMSO vehicle control for 3 days. Viability was determined as a measure of ATP using the CTG assay and dose response curves were generated (Fig. 5d). This is one example of how suspended BME hydrogel cultures can be used directly in a downstream application.

### Suspended BME hydrogel cultures facilitate alternative organoid-derived models

**Figure 6:**
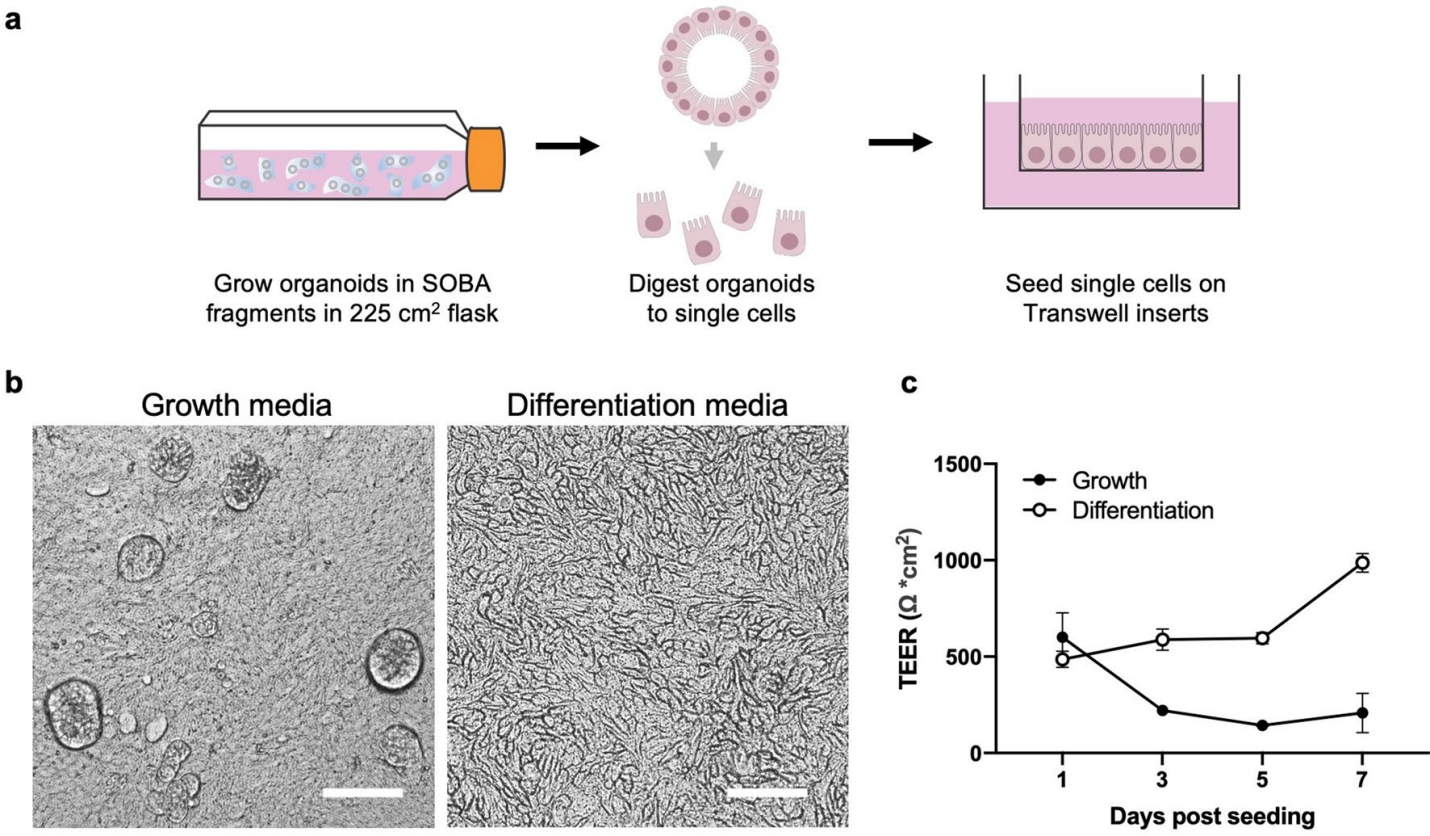
Suspended BME hydrogel organoids to generate colon Transwell epitihelial monolayers. (**a**) Schematic of experiment. SOBA fragment organoid culture in a 225 cm^2^ flask was digested to a single cell suspension, then seeded in 96-well BME-coated Transwell inserts at confluence. (**b**) Brightfield images of SOBA fragment organoid-derived Transwell monolayers 3d post-seeding. Transwells were established with Monolayer Growth Media or Monolayer Differentiation Media. Scale bars are 100 µm. (**c**) Transepithelial Electrical Resistance (TEER) of Transwell monolayers cultured in Monolayer Growth Media (filled circles) or Monolayer Differentiation Media (empty circles). Data represented are mean ± SD, n = 6 wells.

In addition to using organoids directly in their suspended BME hydrogel formats, these organoids can facilitate generation of other organoid-derived models, especially those that require large cell inputs like monolayers and microphysiological system (MPS) devices. We used suspended BME hydrogel organoids to generate colon epithelial Transwell monolayers and evaluated epithelial barrier function in two conditions. SOBA fragment organoids cultured in a 225 cm^2^ flask were digested to single cells, seeded at confluence on 96-well Transwell inserts, and cultured in monolayer growth media or monolayer differentiation media for 7 days (Fig. 6a). The two conditions resulted in Transwell epithelial cultures with different morphologies: in monolayer growth media the resulting epithelium formed 3D structures that protruded from the monolayer surface, while in monolayer differentiation media these structures were absent and a “cobblestone” cell morphology typical of differentiated epithelial cells with mature tight junctions was visible (Fig. 6b). Consistent with this morphology, the epithelial barrier function, quantified by transepithelial electrical resistance (TEER) measurements, was higher for Transwell monolayers in differentiation media than in growth media by day 3 (Fig. 6c). This experiment is an example of how organoids grown using the BOBA, SOBA, or SOBA fragment methods can enable the production of alternative intestinal organoid-derived in vitro models.

## Discussion

Here we describe the development of a suspended BME hydrogel culture method for human intestinal epithelial organoids that overcomes several challenges associated with the conventional surface-attached Dome hydrogel culture method. Compared to the Dome method, the BOBA, SOBA, and SOBA fragment methods simplify and expedite the protocol, enable compatibility with scalable culture vessels, allow kinetic culture sampling, and improve organoid culture uniformity.

Several suspension culture protocols have been proposed for intestinal organoid and tumoroid scale-up, however in these methods organoids were cultured in liquid media containing low concentrations of solubilized Matrigel (similar to BME) which does not form an intact hydrogel^15,16^. This contrasts with our method which relies on the formation of fully cured intact hydrogel. Furthermore, previous studies showed that while organoids and tumoroids can be grown in a 5% solution of soluble Matrigel, concentrations above 5% resulted in reduced growth. Moreover, the intestinal organoid cultures grown in 5% Matrigel exhibited a proportion of organoids with inverted apical-out epithelial polarity or mixed polarity^16^, which is consistent with previous reports of organoid polarity reversal in low ECM conditions^17^. These mixed cultures may not be suitable for many applications, since the apical and basolateral surfaces of epithelial cells are distinct in their composition and biology, and cells often respond differently depending on which surface is exposed to stimuli (e.g., signaling molecules, nutrients, and microbes.).

The BOBA method provides several advantages over the conventional Dome method. First, it simplifies the protocol. Since the hydrogel is cured directly in the media, it eliminates the need for a separate curing incubation step, thus saving time and labor. Next, it is technically more accessible and does not require the precision necessary to plate domes. Furthermore, it increases the culture scalability in several ways. By relieving reliance on available surface area, the BOBA method takes advantage of all 3 dimensions of the culture vessel, resulting in increased hydrogel volume and organoid cells per culture. The BOBA method facilitates substantial culture scale-up because it enables compatibility with flasks, which are available in larger sizes, are easier to handle, and enable rapid media changes. For example, in BOBA culture, 10 mL BME can be cultured in a single 225 cm^2^ flask and media can be changed by simply replacing the supernatant with a serological pipette. However, using the Dome method, nine 24-well plates (200 wells of 50 µL domes) would be required for an equivalent culture and media would need to be changed individually for each well. This also reduces the amount of plastic consumed by over 70% (A 225 cm^2^ flask contains 152.7 g of plastic and replaces 562.5 g of plastic in nine 24-well plates, data not shown). Further scale-up can be achieved using multi-layer flasks or “cell factories” that can accommodate several liters of culture volume. We report general guidelines for suspended BME hydrogel culture setup for several vessel types (Table S1), but users will need to optimize the culture conditions for their specific purposes. Culture vessel type has been reported to impact parameters like gas transfer^18^, and factors such as media formulation, hydrogel composition, and organoid line-specific growth rate can all impact organoid growth. Overall, the BOBA, SOBA, and SOBA fragment methods reduce time, labor, and resources, and thus cost, required to generate large scale organoid cultures.

The BOBA method also overcomes culture heterogeneity that occurs from hydrogel diffusion limitations that occur in Dome cultures^9,10^. Several protocols have suggested plating smaller 10–15 µL hydrogel domes^5,19^, thereby reducing the diffusion path for molecular transport. However, plating smaller domes, even when plating multiple domes per well, reduces the total hydrogel volume per well. Another approach to overcome hydrogel diffusion restriction is to invert the plates during the hydrogel curing step such that gravity causes the cells to settle near the surface of the dome, with few or no cells in the dome core^5^. This results in suboptimal use of expensive hydrogel volume, and ultimately fewer organoid cells that can be seeded per µL of hydrogel. Complex bioengineered approaches have been developed to increase hydrogel surface area to improve molecular transport^10^, but these are difficult to scale up and still rely on anchoring the hydrogel to a 2D surface. The BOBA method generates cultures that are more homogeneous without sacrificing the amount of hydrogel per well or number of cells per µL of hydrogel. The observed improvement in organoid culture uniformity may be explained by the BOBA hydrogels having smaller diameters and thus shorter diffusion paths, and all outer surfaces exposed to the media enabling even molecular diffusion into the hydrogel.

Another documented challenge with organoid morphology heterogeneity in hydrogel domes is that organoids located near the plate bottom can attach to the plate surface, spread and flatten, and lose their 3D structure^5,15^. Since the suspended hydrogel droplets are not in direct contact with the plate surface, organoid spreading and flattening does not occur.

In addition to BOBA hydrogel droplets, organoids can be grown as SOBA filaments and SOBA filament fragments. Organoids grow similarly in all 3 configurations, demonstrating the robustness of the suspension BME culture method. The SOBA method facilitates and expedites culture preparation since a large volume of cell-BME solution can be loaded into a single syringe to produce SOBA hydrogel filaments. About 10 mL of SOBAs can be generated in under a minute, whereas an equivalent Dome culture would require over 15 min to plate and additional 15–30 min to cure. Although the SOBAs are much longer in length than the BOBAs, heterogeneity in organoid growth (like that observed in Dome cultures) was not observed, likely because the SOBA diameters are smaller (typically less than 2 mm) and enable efficient nutrient transport from the media. SOBA fragments more closely resemble BOBAs in size and geometry, but are much faster to generate. Like the BOBAs, the SOBA fragments are evenly dispersed throughout the culture, which can be useful for kinetic sampling or dividing a culture for multiple applications or readouts.

The utility of the suspended BME hydrogel-grown cultures are demonstrated here in two applications. First, SOBA fragment cultures were homogenized to generate 96-well plate cultures with low well-to-well organoid variability. As a proof of concept, a drug toxicity screen was performed; this method can be used in other medium-to-high throughput screens. Second, SOBA fragment organoids could be used to produce organoid-derived Transwell monolayers, which provide the advantage of simultaneous apical and basolateral access, but are difficult to scale up because they require large cell numbers to seed. By enabling substantial organoid expansion, the suspended BME hydrogel culture method can facilitate development and implementation of complex intestinal organoid-derived models with higher tissue fidelity and experimental advantages.

Overall, the suspended hydrogel culture method enables massive organoid scale-up, improves organoid culture uniformity, and saves labor, time and resources. These culture improvements make intestinal organoids more amenable to implementation for high-throughput applications like compound screens or genomic screens. The method can likely be extended to culture diseased intestinal organoids and tumoroids, intestinal organoids from other species, and organoids from different tissue types. Thus, the BOBA, SOBA, and SOBA fragment culture methods have the potential to advance and expand adoption of organoid technology.

## Experimental procedures

### Human intestinal organoid derivation

Organoids were derived as previously described^5^ with some modifications. De-identified human colon and ileum tissue samples from deceased donors were procured by Donor Network West. Tissues were washed with Advanced DMEM/F12 media (ThermoFisher), cut into 5 cm × 5 cm segments, then the epithelium was scraped off from the submucosa into the media and minced with a razor blade. The solution was pelleted at 450 × g for 5 min, then incubated in 2.5 µM EDTA in PBS without Mg^2+^ or Ca^2+^ at 37 °C for 9 min (ileum) or 12 min (colon), with vortexing every 3–4 min until crypts were released. Crypts were pelleted at 450 × g for 5 min, washed in PBS, filtered through sterile gauze then a 100 µm cell strainer to remove debris, and pelleted at 450 × g for 5 min. Crypts were resuspended in Cultrex Reduced Growth Factor Basement Membrane Matrix, Type II (BME, R&D Systems cat. no. 3533-010-02) on ice, plated in 50 µL domes in a 24-well plate, cured at 37 °C for 15–30 min, then overlaid with 500 µL Colon Passage Media (Intesticult Organoid Growth Medium (OGM, StemCell Technologies cat. no. 06010) + 10 µM Y-27632) or Ileum Media (OGM + 10 µM Y-27632 + 2.5 µM CHIR99021). After the first 2–3 days in culture, media was changed every 2–3 days, or when media turned yellow, with plain OGM for colon cultures, or Ileum media for ileum cultures.

### Organoid maintenance

Organoid cultures were passaged every 1–2 weeks by digestion with TrypLE Express (ThermoFisher) at 37 °C for 10 min then trituration with a P1000 pipette. The incubation was repeated up to 2 times if necessary to achieve a single cell suspension. TrypLE Express was inactivated by dilution with PBS, and cells were pelleted at 450 × g for 3 min. Cells were resuspended on ice in BME at 6 × 10^5^ cells/mL, plated in 24-well plates in 50 µL domes and cured at 37 °C for 15–30 min. Domes were overlaid with Colon Passage Media or Ileum media for the first 2–3 days, and media was changed with plain OGM for colon organoids or Ileum Media for ileum organoids every 2–3 days. For colon organoid differentiation, cultures were washed with Advanced DMEM/F12 media, then overlaid with Intesticult Organoid Differentiation Media (ODM, StemCell Technologies cat. no. 100-0214) + 5 µM DAPT for 5 days, with a media change every 2–3 days. Experiments were performed with colon organoids derived from 2 donors and ileum organoids from 1 donor. Variability in organoid growth was observed between donor lines, as well as between low and high passages of the same donor line, however the trends of organoid growth in Dome and BOBA cultures were maintained for different donor lines and passages when compared in parallel within an experiment.

### Suspended hydrogel BOBA culture

Single organoid cells in BME were prepared on ice as described above. Pre-warmed Colon Passage Media or Ileum Media was added to 6-well plates (5 mL/well), 100 cm petri dishes (15–30 mL/dish), or 50 mL conical tubes (30 mL/tube) and kept on a warm bead bath at 37 °C. Ultra-low attachment (ULA) or standard tissue culture plates yielded similar results. To generate suspended BME droplets, or BOBAs, the organoid cell-BME solution was dispensed directly into the warm media in 10 µL volumes using an electronic repeater pipette (Integra VIAFLO 300) with a wide-bore or cut pipette tip (tip opening about 2 mm). The tip was submerged immediately beneath the liquid surface during dispense, then lifted after each dispense to ensure droplet separation. For larger format cultures, BOBAs were transferred into flasks by wide-bore serological pipette or by decanting. Media was changed every 2–3 days with plain OGM for colon organoids or Ileum Media for ileum organoids. In 6-well plate cultures, a sterile 70 µm cell strainer was placed in the well, the plate was tilted and 4 mL media was gently aspirated through the strainer. In flask cultures, the flask was tilted at an angle, BOBAs were allowed to settle in a corner of the flask, then approximately 2/3 volume of spent media was replaced with a serological pipette.

### Suspended hydrogel SOBA and SOBA fragment culture

Single organoid cells in BME were prepared on ice as described above. Pre-warmed Colon Passage Media was added to 6-well plates (5 mL/well), 100 cm petri dishes (15–30 mL/dish) and kept on a warm bead bath. To generate suspended SOBA filaments, the cell-BME solution was gently aspirated into a syringe with a 15-gauge blunt-tip needle, then extruded directly into the warm media while moving the submerged needle in a linear, snake, or spiral motion in the X–Y plane. To generate SOBA filament fragments, SOBA filament cultures were gently triturated twice using a 10 mL serological pipette or wide bore P1000 pipette tip. Additional media was added to bring the final BME-to-media ratio to 1:10. Media changes were performed as described above for BOBA cultures.

### Brightfield microscopy and image analysis

Cultures were imaged by brightfield microscopy using a THUNDER DMi8 inverted light microscope (Leica) with a 2.5×, 4×, or 10× objective, and a DFC9000 GTC camera (Leica). Images were analyzed using Imaris Image Analysis software and the Imaris Batch software package (Oxford Instruments). For automated organoid diameter measurements, the Imaris Surfaces detection module was used with inverted brightfield images. Background and out-of-focus organoids were excluded using a background subtraction step (rolling ball, 19.5 µm diameter). Detected surfaces were then filtered based on four criteria: a software assigned quality metric (> 3000 A.U.), length of the minor axis (> 35 µm—excludes debris), oblong circularity (> 0.2—excludes shadows), and length of the major axis (between 35 and 600 µm, excludes false detection of overlapping organoids). Of the remaining surfaces (at least 40 per image analyzed), we report the diameter as the longest side of the smallest object-oriented bounding box. This process was then run in batch across all analyzed images. Means for each image were calculated, and the mean of three total experiments for n = 3 replicates were plotted.

Spatial organoid uniformity organoid diameter analysis was performed using FIJI (ImageJ). A horizontal rectangle (1.5 mm × 9 mm) ROI was set across the center of each image and divided into 1 mm sections in the X-axis. For each section, diameters were measured manually across the widest point of each organoid.

### Immunofluorescence sample preparation and confocal microscopy

24-well Dome cultures were fixed with 2% paraformaldehyde (PFA) in PBS. Domes were detached from plate using a spatula, and transferred into microcentrifuge tubes using a cut P1000 pipette tip. For BOBA cultures, 500 uL of culture was transferred to a microcentrifuge tube using a cut P1000 pipette tip, the media was removed and 2% PFA in PBS was added. Samples were incubated in fixative at RT for 15–30 min, then washed 3X in PBS. Samples were stained in microcentrifuge tubes with primary antibodies diluted in Blocking/Permeabilization Buffer (3% BSA, 0.1% Triton X-100, 0.02% sodium azide in PBS) for at least 4 h at RT, then washed 3X in PBS. Primary antibodies used are as follows: ɑ-Ki67 (Invitrogen cat. no. MA5-14520), ɑ-MUC2 (Millipore cat. no. MABF1989), ɑ-FABP1 (Novus cat. no. NBP-87695), and ɑ-CHGA (Novus cat. no. NB120-15160). Samples were then incubated with secondary antibodies (donkey ɑ-rabbit Alexa Fluor 488 (ThermoFisher cat. no. A-21206) or goat ɑ-mouse Alexa Fluor 594 (ThermoFisher cat. no. A-11032)), with DAPI, and AlexaFluor 660 Phalloidin diluted in Blocking/Permeabilization Buffer at RT for at least 2 h at RT. Images were collected on a Stellaris 8 Confocal Microscope (Leica) using a 40X objective and 3D-reconstructed using Imaris Image Analysis software (Oxford Instruments).

### Transcriptomic analysis

For RNA isolation, the RNeasy Micro Plus kit (Qiagen) was used (n = 3 wells for each condition). RLT + lysis buffer was added to either BME domes or pelleted BOBAs and stored at − 80 °C. RNA isolation was performed using the QiaCube Connect (Qiagen) and RNA was quantified using a Nanodrop 8000 (ThermoFisher). Bulk mRNA-seq (NovaSeq PE150) and analysis was performed by Novogene. Reads were aligned using HISAT2^20^, differential gene expression analysis was performed using DESeq2^21^, and statistical significance calculated using the negative binomial distribution model with Benjamini–Hochberg FDR correction. PCA analysis was performed using the gene expression value (FPKM) of all samples.

### 96-well plate suspended BME organoid variability

Colon organoid SOBA fragments in a 225 cm^2^ flask were collected after 9 days of culture in OGM and gently triturated twice using a serological pipette to homogenize the sample without disrupting intact organoids. The organoid mixture was transferred to a reagent reservoir and then plated at 100 µL/well in a 96-well plate using a P200 multichannel pipette with wide-bore pipette tips. Dome cultures were prepared as described above and plated in 5 µL domes in the center of each well of a 96-well plate using a repeater pipette. Dome cultures were grown for 7 days in OGM before viability measurements were performed. All viability measurements were performed using the Cell Titer Glo 3D Assay kit (Promega), and luminescence was measured on an Ensight plate reader (Perkin Elmer).

### 96-well plate suspended BME organoid cytotoxicity assay

Two 96-well plates were seeded from homogenized colon organoid SOBA fragments in a 225 cm^2^ flask at 90 µL per well. Compound stocks were diluted to 10× final concentration in Intesticult OGM and 10 µL was added to each well. An 8-dose dilution series with five-fold dilutions starting at 100 µM was evaluated in technical quadruplicates. After 3 days of treatment, viability measurements were performed using the Cell Titer Glo 3D Assay kit (Promega), and luminescence was measured on an Ensight plate reader (Perkin Elmer). 4PL fit curves were generated using Prism (GraphPad).

### Transwell monolayers

Colon organoid SOBA fragments in a 225 cm^2^ flask were collected in a 50-mL conical tube and pelleted at 800 × g for 3 min. Supernatant was removed and organoids were digested to single cells by incubation in TrypLE Express in a 37 °C water bath for 10 min, then triturated with a P1000 pipette. Incubation and trituration was repeated as necessary up to 2×. Cells were pelleted and washed with PBS before resuspension in either Monolayer Growth Media (Intesticult OGM + 10 µM Y-27632) or Monolayer Differentiation Media (Intesticult ODM + 10 µM Y-27632). For each well of a 96-well PET Transwell plate (pore size 0.4 µm, Corning cat. no. 3450), 2.0 × 10^5^ cells in 100 µL of media was seeded in the apical chamber and 200 µL media was added to the basal chamber. Media in both chambers was changed every 2–3 days. Brightfield imaging was performed using a 10X objective and THUNDER microscope (Leica) with a DFC9000 GTC camera (Leica). Transepithelial electrical resistance (TEER) was determined by measuring resistance with a volt/ohm meter (EVOM3, WPI), then multiplying resistance by the Transwell surface area (0.143 cm^2^).

### Statistical analysis

All statistical analyses were performed using Prism 9 software (Graphpad) unless otherwise stated. Statistical tests, n, and p-values are indicated in figure legends.

## Supplementary Information

Supplementary Information.

## Data Availability

The datasets generated and/or analyzed during the current study are available in the National Center for Biotechnology Information Gene Expression Omnibus repository, accession number GSE226539 with token ojglqscyvxszxof.
