# Supplementary material for: Suspended hydrogel culture as a method to scale up intestinal organoids
Source: Sci Rep. 2023 Jun 27;13:10412. doi: 10.1038/s41598-023-35657-9 (PMC10300005; doi:10.1038/s41598-023-35657-9)
Supplement: Supplementary file 1 — Supplementary Information. [file 41598_2023_35657_MOESM1_ESM.pdf]

Supplemental Information

Suspended hydrogel culture as a method to scale up intestinal organoids

Authors

Julia Y. Co[1], Jessica Klein[1], Serah Kang[1], and Kimberly A. Homan[1*]

Affiliations

[1]Complex in vitro Systems, Safety Assessment, Genentech

1 DNA Way, South San Francisco, CA 94080 USA

*Correspondence: homan.kimberly@gene.com

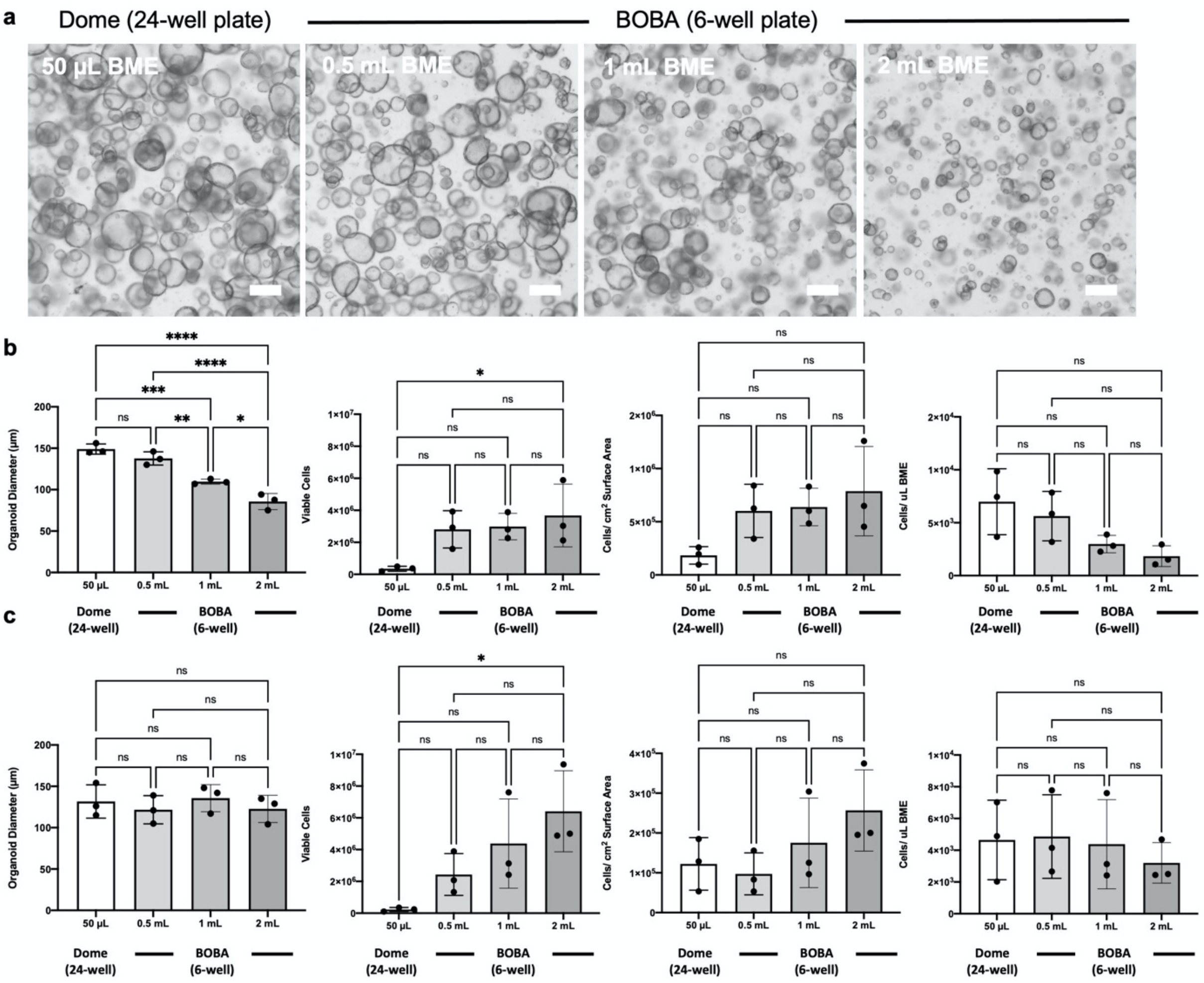

Figure S1. BME volume and culture vessel can impact organoid growth in suspended BME hydrogel culture. (a) Brightfield images of colon organoids in surface-attached Dome in a 24-well plate (50 µL BME in 0.5 mL media), or in BOBA cultures in a 6-well plate well (0.5, 1, or 2 mL BME in 5 mL media). Scale bars are 200 µm. (B, C) Quantification of organoid diameters, total viable cells, viable cells per cm^2 surface area, and viable cells per µL of BME in (b) 6-well plates or (c) 25 cm^2 flasks. Data represented are mean ± SD, One-way ANOVA multiple comparison test, n = 3 experiments; *$p \leq 0.05$, **$p \leq 0.01$, ***$p \leq 0.001$,****$p \leq 0.0001$.

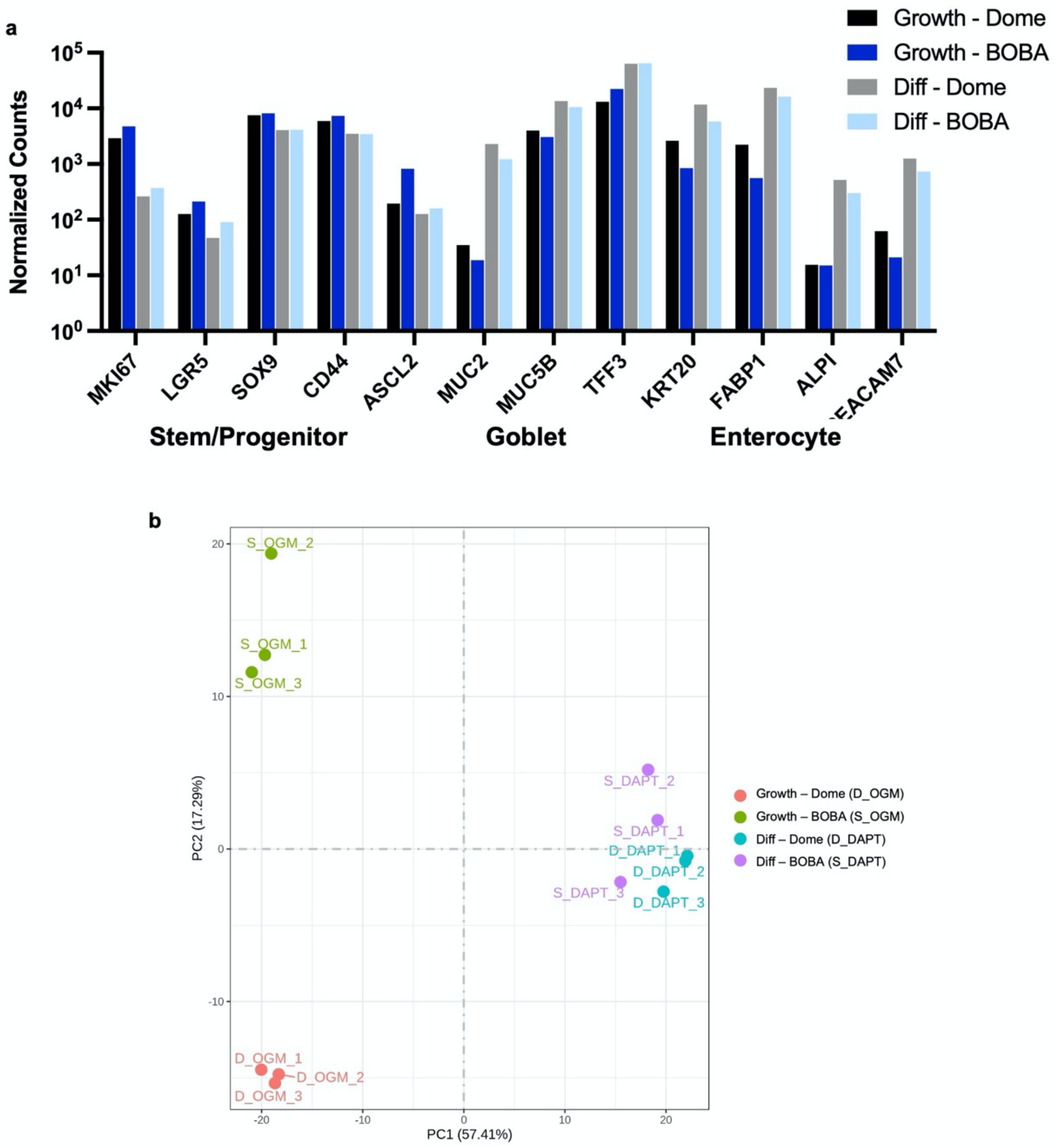

Figure S2. Gene expression of organoids in Dome and BOBA culture. (a) Expression of stem and progenitor cell markers, goblet cell markers, and enterocyte markers of organoids grown using the Dome or BOBA methods, cultured in Growth Media or Differentiation Media. Data represented are mean ± SD for normalized counts determined by DESeq2, n = 3 replicates. (b) Principal component analysis (PCA) plot of Dome or BOBA organoids cultured in Growth Media or Differentiation Media, n = 3 replicates. Separate clustering of BOBA and Dome-grown organoids in Growth Medium may be due to the organoid heterogeneity in Dome cultures.

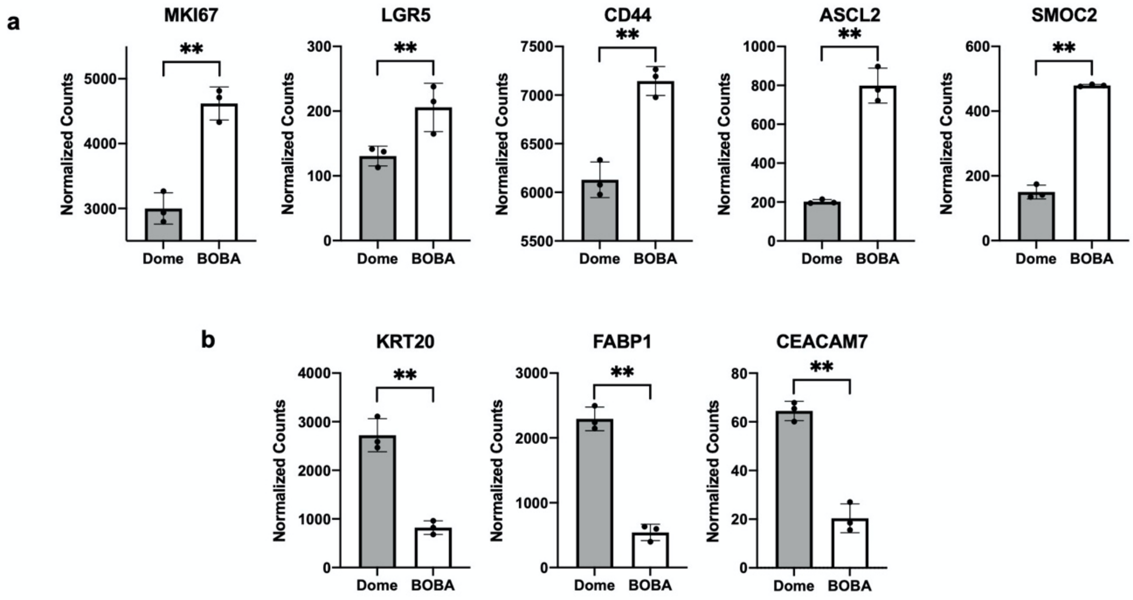

Figure S3. Comparison of gene expression in Dome- and BOBA- cultured organoids in Growth Media. Bulk RNA-seq analysis shows differences in gene expression of (a) stem cell and proliferation markers and (b) enterocyte markers between colon organoids in Dome culture or suspended BOBA culture after 7d culture in Growth Media. Data represented are mean ± SD for normalized counts determined by DESeq2, n = 3 replicates, statistical analysis by the negative binomial distribution model with BH adjusted p-values, *padj ≤ 0.05, ** padj ≤ 0.01.

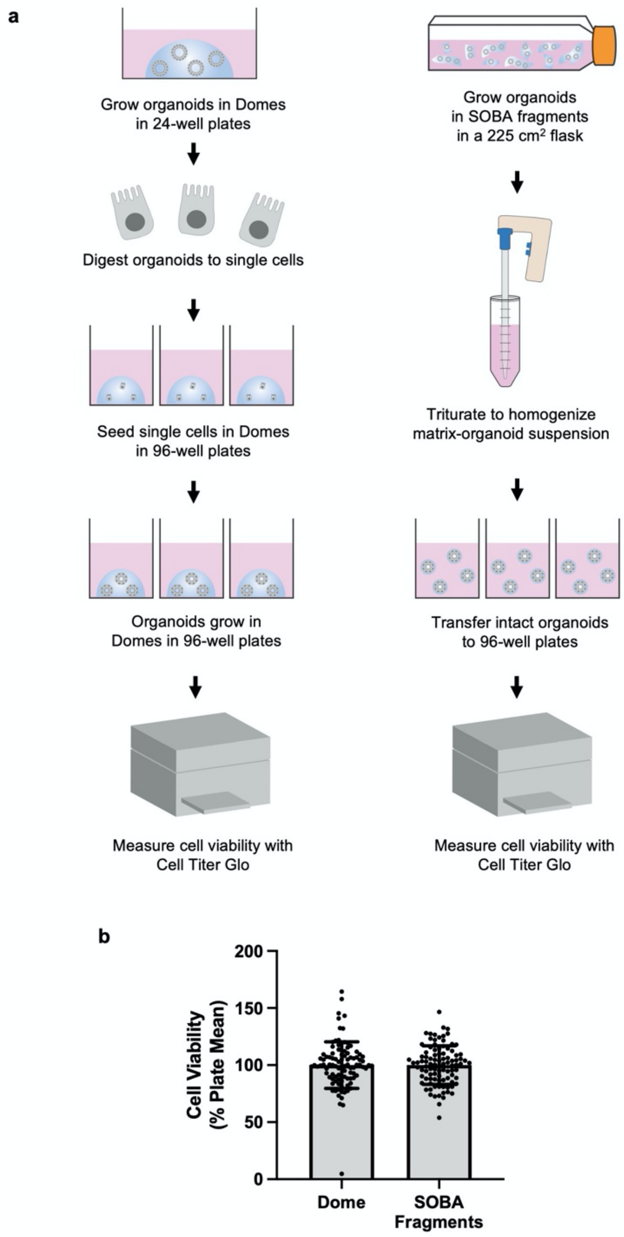

Figure S4. SOBA fragment-grown organoids in a medium-throughput format. (a) Schematic of methods to generate and quantify 96-well plate cultures of organoids grown using the Dome culture method (left) or homogenized SOBA fragment method (right). (b) Cell Titer Glo 3D cell viability readout shows similar well-to-well variability between Dome and suspended BME cultures in a 96-well plate. Each point represents a single well of a 96-well plate (n = 96 wells per condition). Bar graphs represent mean ± SD.

	Surface-Attached BME Domes	Suspended BME Hydrogels (BOBA, SOBA, SOBA Fragments)		
Vessel	24-well plate	6-well plate	75 cm^2 Flask	225 cm^2 Flask
Media per well (mL)	0.5-1	3-5	n/a	n/a
Media per plate/flask (mL)	12–24	18–30	15–30	80–100
BME per well (µL)	30–50	300–700	n/a	n/a
BME per plate/flask (µL)	720–1200	1800–4200	1500–6000	8000-15000
Seeding density (cells/mL)	3×10^5–6.0×10^5	3×10^5–6.0×10^5	3×10^5–6.0×10^5	3×10^5–6.0×10^5

Table S1. Recommended seeding conditions for suspended BME hydrogel cultures. We provide suggested conditions for suspended BME hydrogel culture seeding, however parameters should be optimized for users' specific applications. Factors such as culture vessel type, media formulation, hydrogel composition, and organoid line can all impact organoid formation and growth rate.
